# Popcorn in the pain clinic: A content analysis of the depiction of patients with chronic pain and their management in motion pictures

**DOI:** 10.1080/24740527.2022.2123308

**Published:** 2022-10-27

**Authors:** Karim Mukhida, Sina Sedighi, Catherine Hart

**Affiliations:** a Department of Anesthesiology, Pain Management and Perioperative Medicine, Faculty of Medicine, Dalhousie University, Halifax, Nova Scotia, Canada; b Faculty of Medicine, Dalhousie University, Halifax, Nova Scotia, Canada; c Novagevity, Halifax, Nova Scotia, Canada

**Keywords:** chronic pain, film, Hollywood, cinemeducation

## Abstract

The watching of films is popular and accessible to broad segments of the population. The depiction of medical conditions in films has the potential to affect the public’s perception of them and contribute to stereotypes and stigma. We investigated how patients with chronic pain and their management are depicted in feature films. Films that contained characters with or references to chronic pain were searched for using databases such as the International Movie Database. Themes that emerged from the content analysis revolved around the films’ depictions of characters with pain, their health care providers, and therapies for pain management. Patients with chronic pain were depicted in various ways, including in manners that could elicit empathy from audiences or that might contribute to the development of negative stereotypes about them. The attitudes of health care professionals toward patients with chronic pain ranged from compassionate to dispassionate. Pain management was typically depicted as lacking in breadth or using multidisciplinary approaches with a focus on pharmacological management. The variety of topics related to chronic pain depicted in feature films lends to their use in medical education strategies to better inform health care professions trainees about chronic pain management.

## RÉSUMÉ

Le visionnement de films est populaire et accessible à de larges segments de la population. La représentation des affections médicales dans les films est susceptible d'affecter la perception qu'en a le public et de contribuer aux stéréotypes et à la stigmatisation. Nous avons étudié comment les patients souffrant de douleur chronique et leur prise en charge sont représentés dans les longs métrages. Les films qui contenaient des personnages ou des références à la douleur chronique ont été recherchés à l'aide de bases de données telles que l’International Movie Database. Les thèmes qui sont ressortis de l'analyse du contenu tournaient autour des représentations des personnages souffrant de douleur, de leurs prestataires de soins de santé et des traitements pour la prise en charge de la douleur. Les patients souffrant de douleur chronique étaient représentés de diverses manières, y compris de manière à susciter l'empathie du public ou contribuer à l'apparition de stéréotypes négatifs à leur sujet. L'attitude des professionnels de la santé à l'égard des patients souffrant de douleur chronique allait de la compassion à la circonspection. La prise en charge de la douleur était généralement décrite comme manquant d'envergure ou utilisant des approches multidisciplinaires, tout en mettant l’accent sur la prise en charge pharmacologique. La variété des sujets liés à la douleur chronique présentés dans les longs métrages favorise leur utilisation dans les stratégies d'éducation médicale pour mieux informer les professionnels de la santé en formation sur la prise en charge de la douleur chronique.

## Introduction

Films are highly accessible and popular, reach broad segments of the population, and remain an important source of entertainment as well as distraction and relaxation.^1–7^ It was recognized even within the first few decades after the first screenings of projected cinematographic motion pictures that films could not only showcase societal preoccupations but also influence public perceptions and behaviors. In 1936, Alexander Markey, then executive producer of the Motion Picture Foundation of the United States, appreciated that films were “revolutioniz[ing] the whole trend of modern mass thought.”^8(p725)^ Cinema’s “primary importance,” he wrote, “lies in its power to shape the social, moral, and cultural values of today—and tomorrow.”^8(p726)^ Bandura’s work on social learning theory has suggested that behaviors are influenced by depictions of reality in the media because “a great deal of information about human values, thinking patterns, and behaviours is gained from models portrayed symbolically through verbal or pictorial means.”^9–11(p66)^ Films in particular have been shown to have the ability to influence attitudes and behaviors regarding political issues,^12^ gender stereotypes,^13^ unsafe sexual activities,^14,15^ and substance use.^16–24^

Public perceptions of and attitudes toward medicine could therefore be affected by their depiction in films. As Gans^25^ wrote, because films are a part of the entertainment business, depicting health care providers and medical conditions realistically is often not the primary goal:

Unlike, say, the supermarket business, which must reflect the demands and wishes of the general public, entertainment caters to a set of specific and fickle audiences, and has to be, virtually by definition, deviant, daring, and even oppositional to the values of these audiences. Popular movies … are not about everyday marriage but about passionate or violent affairs; they do not deal with car theft but with murder; they ignore life in the suburbs for life in the Mafia.^25(p151)^

It is not surprising, then, that physicians and medical conditions have been shown in stereotypical ways. Flores^4^ found that physicians were portrayed negatively in almost half of the 131 films they reviewed. Medical conditions have also been shown stereotypically, with psychiatric conditions such as psychosis, for instance, being associated with criminality and violence.^26,27^ Certain characteristics of medical conditions or their treatments may be emphasized and others minimized or they may not be presented realistically.^2^ The concern has been that such characterizations may be “processed unintentionally and unconsciously” by the audience and influence their attitudes and perspectives about medicine.^28(p276)^ In the case of physician portrayals, this has been argued to have the potential to adversely affect the therapeutic relationship between physicians and patients, such as by causing distrust of physicians or even trepidation about seeking care.^4,28,29^ In the case of medical conditions, there are concerns that films will reinforce stereotypes and perpetuate stigma and discrimination,^2,26,27^ as was seen to be the case with public attitudes to electroconvulsive therapy after its depiction in *One Flew Over the Cuckoo’s Nest*.^30^ Such concern has been recognized by the film industry, with organizations like the British Academy of Film and Television Arts promoting programs that support greater public understanding of mental health issues.^29,31^

Although the depiction of a variety of medical professionals, conditions, and procedures in films has been studied, to date there have been no studies focusing on the portrayal of characters with chronic pain or their management in films. Mueri and colleagues^32^ looked at the portrayal of pain in movies and television shows, but their study focused only on media targeted toward 4- to 6-year-old children, and in these portrayals over 99% of instances of pain were related to acute injuries or violence. The aim of this study was therefore to explore portrayals of chronic pain and its management in films. No hypotheses were made a priori because it was not certain how much content existed or how it would be portrayed, although based upon the results of studies examining the portrayal of other medical conditions in film, it was thought that this might occur in a manner that stigmatizes patients with chronic pain or its management.

## Methods

A qualitative content analysis was performed. First, a list of films was compiled that featured characters with chronic pain or the management of chronic pain independently by two reviewers who searched through the International Movie Database (IMDb; https://www.imdb.com), the Literature Arts Medicine Database (https://medhum.med.nyu.edu/about), the History of Medicine and Medical Humanities Databases (https://medhumanities.mcmaster.ca/index/mcmaster-library-collections/resources-themes-bibliographies/history-of-medicine-and-medical-humanities-database), and Medicine on Screen (https://medicineonscreen.nlm.nih.gov/category/all/). IMDb, an online database of over 8 million films, television series, and streaming online content from 1888 to the present time, is considered a validated tool to search for films for research purposes.^2,33–36^ The following search terms for keywords were used: “pain” and “chronic pain.” These terms were purposefully kept broad to increase the probability that any relevant films were found. The databases were queried for films between January 1980 and January 2022. Rather than reviewing an “exhaustive” list of films that make any reference to chronic pain, representative films were considered.^4(p657)^

Inclusion criteria included films that depicted characters with chronic pain. The International Association for the Study of Pain’s definition of pain as “an unpleasant sensory and emotional experience associated with, or resembling that associated with, actual or potential tissue damage” was used to select films related to pain.^37^ Cues from the films in terms of each film’s timeline or references made by film characters with respect to pain were used to determine the chronicity of the pain with the aim of selecting films in which the duration of pain seemed to be of at least 3 months’ duration, consistent with chronic pain’s classification as per the 11th revision of the *International Classification of Diseases*.^38^ Only films released after 1980 were retained because we were interested in films that might contribute to contemporary attitudes about chronic pain. Films had to have had a theatrical release or be obtainable on either YouTube, Netflix, AmazonPrime, iTunes, or Amazon because included films needed to be those that could be easily accessible to the public. Any films whose focus was acute pain or its management were excluded from review. Also excluded were children’s movies and animated films because recent studies have already looked at aspects of their depiction of pain.^32^ Documentaries and any television or film series were excluded, as were internet and direct-to-video films. Following the method of Auwen and colleagues,^2^ we included films that were deemed likely to be associated with “significant cultural impact” from a North American perspective because they might be more likely to influence public perceptions in North America. We therefore sought films that were produced by Hollywood film companies or that had a connection to Hollywood via nomination for Hollywood-associated awards. Measures to assess such significance include films’ receipt of awards, significant box office revenue, and/or favorable reviews. Films that were nominated for or received Academy or Golden Globe awards, earned more than $50 million of box office revenue (www.the-numbers.com, IMDb, or www.boxoffice.mojo), and/or received favorable reviews by critics as reported by their Metascore (>75; Metacritic) or Tomatometer (>75; www.rottentomatoes.com) met the inclusion criteria.

The compiled films were viewed independently in their entireties by two reviewers (K.M. and S.S.). Dialogues related to chronic pain or its management were transcribed and scenes were described by these reviewers. A qualitative content analysis of the films was performed using approaches described by others for their film analyses.^4,6,13,28,39^ After the films were watched, each reviewer (K.M. and S.S.) transcribed the scenes that they thought were relevant for analyses. The reviewers then met to compare their transcripts for each film. A coding template was created after initial discussion. Ideas for themes related to how characters with chronic pain and the management of chronic pain were portrayed were discussed together and organized into themes based upon consensus. This was an iterative process.

## Results

**Figure 1.**
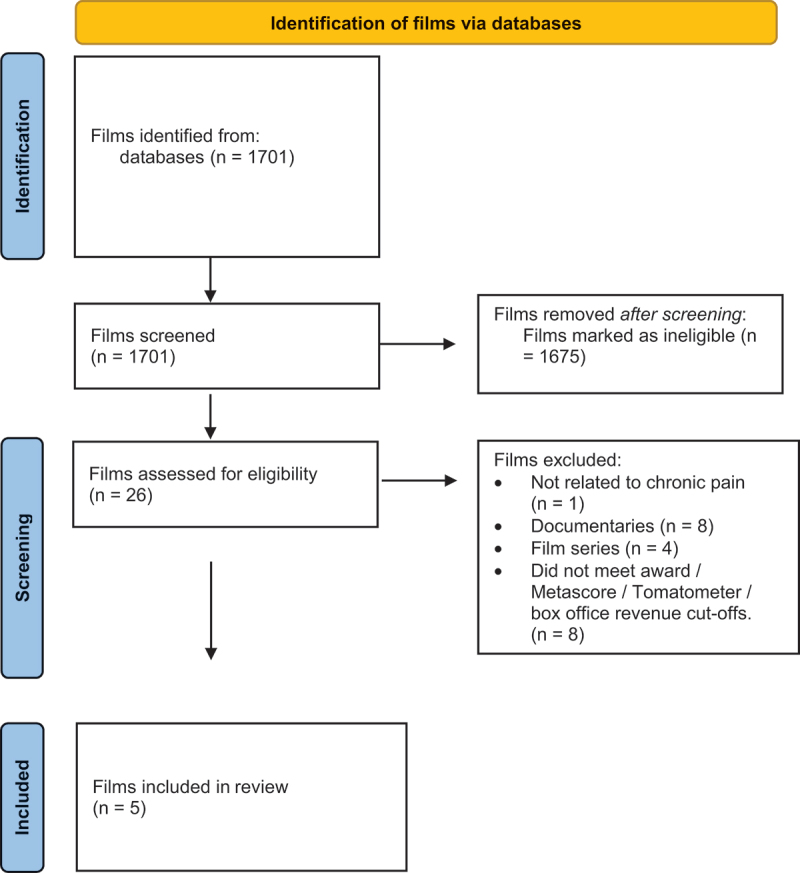
Article management search process. Adapted from Page and colleagues.^129^

The search strategy identified five films for review and analysis (Table 1, Figure 1). All were dramas that were made for cinema. Films that were excluded after assessment for eligibility were those that were series (*n* = 4) or documentaries (*n* = 8) or that did not meet the inclusion criteria in terms of receipt or nomination of awards, earning of box office revenues, or favorable enough reviews (*n* = 8). Upon closer review, one film did not deal with chronic pain.

**Table 1.** Films analyzed for depictions of chronic pain and its management.

Film	Year of release	Box office earnings ($)	Metascore	Tomatometer (%)	Selected awards won
*The English Patient*	1996	231,976,425	87	85	Academy Awards: Best Picture, Best Director, Best Supporting Actress, Best Art Direction, Best Cinematography, Best Costume Design, Best Film Editing, Best Original Dramatic Score, Best Sound
					British Academy of Film and Television Arts: Best Film, Best Actress in a Supporting Role, Best Cinematography, Best Editing, Best Original Music
					Golden Globes: Best Motion Picture–Drama, Best Original Score–Motion Picture
*Frida*	2002	56,298,474	61	75	Academy Awards: Best Makeup, Best Original Score; nominations for Best Actress, Best Art Direction, Best Costume Design
					American Film Institute Awards: Top Ten Movies of the Year
					British Academy of Film and Television Arts: nomination for Best Actress
					Golden Globes: nomination for Best Actress
					Golden Globes: nomination for Best Foreign Language Film
*Cake*	2014	2,433,850	49	49	Golden Globes: nomination for Best Actress–Motion Picture Drama
*The Upside*	2017	125,856,180	46	43	
*Pain and Glory*	2019	37,359,689	87	96	British Academy of Film and Television Arts: nomination for Best Film Not in the English Language
					Cannes Film Festival: Best Actor; nomination for Palme D’Or
					Golden Globes: nominations for Best Foreign Language Film, Best Actor–Motion Picture Drama

A variety of themes emerged from the qualitative content analysis. These are broadly categorized into themes related to the depiction of

1. characters with chronic pain:

(a) chronic pain as adversely affecting multiple dimensions of characters’ lives as well as affecting the lives of those close to them;

(b) characters with chronic pain may have considered themselves or were portrayed as being a burden to others, or

(c) in unfavorable ways; and

(d) chronic pain as being an unbearable experience leading to suicidal ideation.

2. health care professionals looking after characters with chronic pain:

(a) health care professionals were out of tune with the needs of characters with pain;

(b) health care professionals provided holistic and compassionate care to characters with pain.

3. therapies for pain management:

(a) opioid and cannabis use were depicted in ways that promote stigma;

(b) the use of the arts to manage pain was favorably portrayed.

### Depictions of Characters’ Lives with Pain

*Chronic pain adversely affects multiple dimensions of characters’ lives*

The way in which chronic pain affects multiple dimensions of patients’ lives was made evident in films in which the protagonist suffered with chronic pain. *Pain and Glory* focuses on the life of a Spanish film director named Salvador Mallo. He is first met floating in a pool, his well-healed midline spine incision visible. Salvador describes suffering from both physical pains as well as “*dolores del alma*” (10:37). As he lists his various pains against a backdrop of anatomical images, he outlines the pervasive influence that chronic pain can have on patients’ sense of selves and worldviews:

I started to know my body through pain and illnesses. I lived my first thirty years pretty much in the dark, but soon I discovered that my head, and what was inside it, besides being a source of pleasure and knowledge, held infinite possibilities for pain. Soon I found … all kinds of muscular pain: lumbar, dorsal, tendinitis, both knees and shoulders. … Headaches are my specialty. Migraines, tension or cluster headaches, and back pain. From my lumbar spinal fusion surgery, which immobilized more than half of my back, I found that my life would revolve around the spinal column. … But not all is physical and illustratable. I also suffer from abstract sorrows, aches of the soul, like panic and anxiety, that add angst and horror to my life. And of course I have toggled with depression for years.

On the nights where several pains coincide, those nights I believe in God. And I pray. On the days that I only have one kind of pain, I’m an atheist. (10.37)

Over the course of the film, the manner in which chronic pain affects his life becomes apparent: there is a stiffness to his movements and he is fatigued. He keeps his apartment dark when he has a headache. He has difficulty sleeping, still lying restlessly in his bed at 4:06 am (38:45). His friend Mercedes recognizes the effect that the pain is having on his ability to perform his instrumental activities of daily living and speaks with his housekeeper about this:

Mercedes: Maya, tell him you don’t mind tying his shoes or his tennis shoes. It must be hard for him to tie his own shoelaces. Poor thing.

Maya: Sure, I tell him that, Mrs. Mercedes. But he doesn’t want me to. I think he’s embarrassed, and I feel sorry for him. I don’t know what to do.

Mercedes: Well, smile and take care of him. And if you see something strange, call me.

Maya: Mrs. Mercedes, everything’s strange here. (39:33)

Chronic pain has adversely affected Salvador’s ability to continue working as a film director. In a chance meeting with an actress that he knows, a sense of loss related to his inability to work is hinted at:

Zulema: I’m surprised it’s you who’s not working. I always thought you’d be the one to never retire.

Salvador: Same here. (6:57)

Mercedes: [encourages Salvador to do more activities to occupy his time, but not being able to do the work that he loves causes him a profound sense of loss]: You should do something. You have too much free time to think about your illnesses. Give your brain something to entertain itself.

Salvador: I’d like to do some things, too, Mercedes [shrugging].

Mercedes: You could write. You have a lot of documents full of ideas to develop. I’ll make you a list.

Salvador: I don’t want to write if I can’t film it. You know better than anybody that I can’t face a shoot in this state. Without filming, my life has no purpose. But that’s just how things are. (49:27)

In *Cake*, viewers similarly obtain longitudinal exposure over the course of the film to the protagonist’s life with chronic pain. *Cake* follows Claire Bennett, a woman suffering with chronic pain after a motor vehicle incident. Claire no longer works as an attorney in Los Angeles and is separated from her spouse. Her body bears physical evidence of trauma with scars on her face and limbs: “What happened to your face?,” a boy asks Claire when he sees her for the first time (53:12). Her actions demonstrate how painful the performance of even mundane tasks and simple movements is. Getting out of a vehicle takes great effort as Claire pulls herself up from lying on her back on the passenger seat of a vehicle, trying not to turn her head and neck too much as she passes money to the taxi driver and climbs out (3:41). Movements require piecing together slow, deliberate, careful acts. She holds her body stiffly as she walks, gently bracing herself on a wall (4:06), or she winces as she climbs onto a bar stool in her kitchen or even just looks down at her phone (4:26). A restful sleep is a but a dream: she tosses and turns and moans with discomfort (6:30). Even a scene of physical intimacy sees Claire holding back tears and bracing herself because of pain (13:44). With a physiotherapist, the pain that Claire experiences is not only seen via her body language but is heard as well: “Goddamn, it hurts!” she screams during an aqua therapy session as the physiotherapist supports her in the water (19:53). Chronic pain thus is shown as having an all-encompassing effect on Claire’s life, including affecting her ability to work and relationships with others.

A sense of pain representing a betrayal of the body was elicited in the films. In *Frida*, Frida Kahlo is depicted as striving to fulfill her life as best she can through painting despite her pain. After Leon Trotsky climbs with her to the top of a pyramid in Teotihuacan (1:29:52), he asks Frida how she was hurt: “I couldn’t even tell you anymore,” she tells him (1:30:06). “I’ve been cut, rebroken, reset so many times, I’m like a jigsaw puzzle. And all the operations have done more damage than the accident for all I know. Everything hurts … but the leg, the leg is the worst” (1:30:08). Despite her resilience, the films shows that her chronic pain detrimentally affects her life and fulfillment; to her lover Diego Rivera she describes her body as betraying her and laments that her pain has even changed how she sees herself:

Frida: I want you to burn this Judas of a body. I don’t want to be buried. I’ve spent enough time lying down. Burn it.

Diego: Frida.

Frida: I don’t think I’m Frida. I think all the Frida in me has disappeared. Look at what’s left. (1:50:35)

Some films also provide the perspective of the effect of characters’ chronic pain on the lives of those around them. For example, in *The English Patient*, Canadian army nurse Hana volunteers to care for the Hungarian cartographer Count László de Almásy. Having suffered with personal losses during World War II, the full-time nursing care that the count requires, including his pain management, provides Hana with both a sense of purpose and distraction.

*Characters with chronic pain considered themselves or were portrayed as being a burden to others*

The way that characters’ pain affected others also provided the impression that they considered themselves to be burdensome. In several films, characters’ pain conditions render them with difficulties mobilizing and looking after themselves. For example, confined to her bed at the beginning of *Frida*, Frida Kahlo feels that her condition has encumbered her family. She responds thus when her father asks her how she is feeling:

Frida: How am I feeling? I can’t even remember what it felt like before the pain. Isn’t that horrible?

Father: Dr. Farill is coming on Monday. He’s bringing a back specialist, Dr. Cervantes.

Frida: I feel like some rich girl with a new suitor every week.

But all my suitors have turned into doctors. I’m not a rich girl, Papa. How come you never ask me about my plans anymore? You used to always say … “Tell me your plans, Frida!”

Father: What are your plans, Frida?

Frida: Right now, I’m a burden. But I hope to be a self-sufficient cripple one day. After that, I don’t know.

Father: You are not a burden, my love. (17:10)

In other films, the characters’ pain and living conditions are such that they require help with their daily activities. In *Cake*, Claire requires significant help with her life. She is supported by Silvana, who looks after her house, prepares meals, and drives her to appointments. In *Pain and Glory*, Salvador benefits from Maya’s help around his apartment. Count Almásy is completely bedbound and dependent upon Hana for all of his activities of daily living secondary to his painful burn injuries in *The English Patient*. He requires her to turn him regularly to prevent bed sores, feed him, and change the orientation of his bed so that he can experience a better view. His contractures have affected his ability to use his hands to the extent that he cannot even grasp a vial of morphine and at best can only use the back of his hand to push it toward Hana to encourage her to open it and draw it up for him for administration (2:29:30).

*Characters with chronic pain were portrayed in unfavorable ways*

Though the depiction of the suffering associated with chronic pain could thus elicit a sense of empathy from the audience, in some films characters with chronic pain were depicted to be manipulative. Count Almásy, for example, behaves in manners that could be considered negatively. He has an affair with Katharine Clifton that causes such heartbreak to her spouse Geoffrey Clifton that he tries to kill all of them in a murder–suicide. The count assists the Germans by providing them with maps of North Africa that enable them to invade Cairo and indirectly lead to the torture and maiming of David Caravaggio, a Canadian spy. Caravaggio has been seeking revenge and believes that the count is responsible not only in his own misfortune but also others’: “You didn’t kill the Cliftons?” he asks the count (2:07:55). “Maybe I did” (2:08:32).

*Cake* opens with a scene in which Claire disrupts the women’s chronic pain workshop by making other participants upset. She “manipulates” a physician who is not aware of her case to renew a prescription for opioids (09:35). She threatens to pursue legal action against the City of Los Angeles to convince the pain workshop leader to give her Nina’s home address (25:45). She concocts a story to convince Nina’s husband to allow her into his home (27:26). Other characters in the film express their frustrations with Claire. For example, Silvana’s daughter feels like Claire is taking advantage of her mother’s kindness. She calls Claire a “bitch” (16:00) and tells her mother that Claire “doesn’t pay [her] enough to put up with her shit” (16:10); “If I were you I would just quit,” she recommends (16:14). Toward the end of the film, after Silvana encourages Claire to get off the train tracks before an oncoming train arrives, Silvana expresses that she is more than just frustrated with Claire:

I’ve been trying to deal with your bad attitude, your insults, but I can’t take it anymore. You sleep with any low-life who walks in front of you, you get drunk, you use drugs … and on top of it all you treat me like a dog, and pay me like a dog! I don’t know why I worry about you. I don’t know why I light candles to the Virgin Mary and ask her to protect you. You drove Mr. Benett away when all he wanted was to take care of you. I’m not frustrated, *señora*! Frustrated is too small a word to describe what I’m feeling. (1:22:26)

Claire has insight into those aspects of her that make her unlikable. “Sometimes I suspect that you think that I’m this uncooperative old bitch who’s just making all this up,” she tells her pool therapist (1:00:57). When a vision of Nina tells Claire that she “just use[s] people” Claire responds, “I know” (51:52), and in a conversation with Jason, Nina’s husband, refers to herself as an “evil witch” (41.56). Even Nina, who is only seen in the film as a vision in Claire’s mind, is viewed by some characters unsympathetically. Claire describes the aftermath of Nina’s suicide in a way that emphasizes the hardship it must have imparted upon her family and friends, and from Nina’s husband we hear how he regards his wife’s suicide as a selfish act.

*Chronic pain as being an unbearable experience leading to suicidal ideation*

That living with chronic pain can be such an overwhelming burden that it can lead to suicidal ideation was another theme raised in some films. Sitting in a wheelchair on his New York City penthouse balcony with his “life auxiliary” caregiver Dell Scott in *The Upside*, Philip Lacasse describes the neuropathic pain that he experiences secondary to a spinal cord injury that has rendered him quadriplegic as “like being on fire” (47:02). “Hurt enough to make you wanna off yourself?” Dell asks (47:20). *Cake* opens with a scene showing a discussion that members of a women’s chronic pain workshop are having about the recent suicide of one of the group members, Nina. Claire has recurrent visions and thoughts of Nina’s suicide: she sees images of Nina jumping off a freeway (21:20), speaks with the highway patrol worker who had witnessed Nina’s suicide (23:13), and goes to the place on the freeway where Nina had jumped and peers over the edge of the freeway to look at the roads and speeding cars below (24:37). It is not just thoughts of Nina’s suicide that preoccupy her but thoughts of her own suicide as well. She dreams that she is drowning in her backyard swimming pool (1:11:19). She pictures herself standing at the same place where Nina had stood on the freeway and falling off the edge (25:31). Her thoughts become manifest in actions: after one of her aqua therapy sessions at a swimming pool, when there is no one else present, she holds onto weights and tries to stay underwater (22:01). While going to a drive-in movie with Silvana in Riverside, she leaves the vehicle and finds an opening through the chicken-wire fence to gain access to train tracks (1:19:31). She lies on the tracks with her head resting on one of the rails with a vision of Nina lying next to her. The vision of Nina suggests that Claire contemplates her own suicide, with Nina at times urging her to follow through with her suicidal ideation: “What’s stopping you? You don’t believe in God, or Heaven, or Hell. You don’t believe in anything … do it [commit suicide] right now … just do it … why don’t you just do it? … don’t be such a coward!” (18:50).

### Depiction of Health Care Professionals Looking after Patients with Pain

*Health care professionals were out of tune with the needs of characters with pain*

Tension can be seen in the relationship between health care professionals and characters with pain. For example, in *Cake*, Claire and her pool therapist struggle to work with each other. Annette, the women’s chronic pain workshop leader, leaves a message on Claire’s voicemail that she is no longer welcome to the group. A pharmacist in Mexico has more of an unethical transactional rather than therapeutic relationship with Claire. He provides Claire with opioids without a prescription and in a manner to facilitate her smuggling them back to California. When Claire expresses concerns about this, the pharmacist points out her privilege: “You’re a rich white woman. Have you ever been caught at anything?” (33:57). He bluntly points out that Claire has “problems” and has little empathy for her.

In some of the films reviewed, physicians appear as not being attuned to the needs of the characters with pain. Physicians appear to be not well-prepared for therapeutic encounters. The physician in *Cake* is not Claire’s primary physician, which would make it even more prudent for her to better acquaint herself with Claire’s history of pain and use of opioids, but she does not. She appears disorganized: “Gosh. I can’t seem to find the authorization from your primary. … I’d lose my head if it wasn’t attached to my body. … I mean, I write reminders about my reminders” (9:52). She becomes distracted during the conversation and proceeds to prescribes Claire Percocet and OxyContin nevertheless: “Just don’t tell anyone,” she asks Claire (10:44). Her comments at the end of the clinical encounter again demonstrate the disconnect between the physician’s interpretation of the patient’s clinical status and reality. Her comments to Claire to “keep up the good work” (10:52) and “you’re doing really great” (10:54) seem so inappropriately juxtaposed with the reality of Claire’s situation that the audience has become aware of—Claire has just been seen struggling with any sort of physical activity and has been kicked out of the pain workshop group for disruptive behavior and in the scene following the visit with the physician is seen taking opioids with alcohol.

There are scenes in which physicians are not seen but disparate remarks are made about them by characters in the films. Frida refers to her doctors as “suitors” and makes reference to the operations that they performed as “hav[ing] done more damage than the accident” (1:30:15). In *Pain and Glory*, Salvador complains to Mrs. Mercedes that he is “sick of doctors” (46:36). Indeed, he therefore obtains oxycodone from a friend whose mother is a pharmacist.

*Health care professionals provided holistic and compassionate care to characters with pain*

An exception to this negative depiction of physicians is seen in *Pain and Glory* after Salvador decides to stop using heroin and he brings Mrs. Mercedes with him to meet with a pain physician. Dr. Galindo appears attentive, empathetic, and professional and his focus is the patient (1:20:11). The physician expresses no judgment when Salvador confesses his use of heroin and his questions seem objective and genuine in assessing Salvador’s readiness to discontinue its use. He looks into the circumstances regarding Salvador’s heroin use and asks about his creative projects, which contribute to the development of a therapeutic relationship.

The provision of pain care by nurses was shown in *The English Patient* and portrayed in a positive light. Hana recognizes her patient’s suffering and confronts ethical dilemmas related to their care by acting to relieve that suffering. Toward the end of *The English Patient*, Hana is depicted as so in tune with her patient that they can converse about serious decisions even through gaze and gestures. As Hana prepares his morphine for pain management, he broaches the topic of his mortality:

Almásy: I’m still here.

Nurse Hana: You’d better be.

Almásy: Don’t depend on it, will you? That little bit of air in my lungs, each day it gets less and less, which is all right. It’s quite all right. I’ve been speaking to Caravaggio, my research assistant. He tells me there’s a ghost in the cloisters. I can join him. (2:27:17)

As Hana breaks the tops off the vials of morphine, Almásy knocks over a box containing more vials and looks at her. He nudges an extra vial toward her, then another two vials, and another two. Hana looks back at him and he looks away. “Thank you,” he says (2:30:34). Hana begins to cry. “Read to me, will you? Read me to sleep,” says Almásy, and Hana nods and cries as she prepares the syringe to help him die and relieve his suffering (2:30:47).

Moreover, in a broader perspective, Hana was seen as holistically providing compassionate care that extended beyond her patient’s pain management:

Nurse Hana: I should try and move the bed. I want you to be able to see the view. It’s good. It’s a view from a monastery.

Almásy: I can already see.

Nurse Hana: How? How can you see anything?

Almásy: No, no, not the window. I can’t bear the light anyway. I can see all the way to the desert.

Nurse Hana: I’m turning you.

Almásy: Exploring before the war, making maps. Is there sand in my eyes? Are you cleaning sand from my ears?

Nurse Hana: No sand. That’s your morphine speaking.

Almásy: I can see my wife in that view.

Nurse Hana: Are you remembering more?

Almásy: Could I have a cigarette?

Patient Hana: Are you crazy?

Almásy: Why … Why are you so determined to keep me alive?

Nurse Hana: Because I’m a nurse. (25:11)

### Depiction of Therapies for Pain Management

*Opioid and cannabis use were depicted in ways that promote stigma*

Opioids are the most common medication used for pain management in the reviewed films. In *The English Patient*, as Count Almásy watches as Carravagio prepares to inject morphine, he acknowledges that he has “come to love … that little tap of the fingernail against the syringe” (44:22). Morphine is later shown to be used to provide the ultimate end to the count’s pain via its facilitation of euthanasia. To deal with her pain, Claire is seen to primarily use opioids such as oxycodone in *Cake*.

Opioid use is sometimes portrayed as being problematic. Claire demonstrates a number of opioid aberrant behaviors. She concurrently takes opioids while drinking alcohol. She manipulates a physician to prescribe opioids for her. It appears that she is taking more opioids than prescribed because she has empty pills bottles and travels to Tijuana to illegally obtain more. Taking too many leads to her overdose and admission to hospital. Salvador first tries heroin because he’s “curious” but then uses it more frequently to manage his pain, obtaining it from Alberto, an actor with whom he had worked in the past, as well as buying it from a drug dealer on the street. Heroin use causes Salvador to be sedated and dysfunctional.

Cannabis is portrayed as having a beneficial effect on pain but is also depicted in a manner that suggests stigma regarding its use in *The Upside*. After learning of Philip’s neuropathic pain, Dell suggests that he try smoking cannabis. “It’s not going to help,” Philip declares (48:12). “Tell me after you try it,” Dell tells him after taking a puff of the joint himself and bringing it to Philip’s mouth (48:15). “You’ll feel it,” Dell tells him confidently. “Wanna bet?” Philip retorts (51:25). The next scene shows Dell vindicated as Philip is seen laughing as he tells Dell, “You should have bet more than hot dog!” (51:31). There is still a sense of stigma related to cannabis use, however, as Dell brings Philip to a secluded part of Central Park for him to try cannabis. It is dark, there is hardly anyone around, and police sirens can be heard in the background, giving the sense that cannabis use is not acceptable enough to be used openly.

*The use of the arts to manage pain was favorably portrayed*

*Frida* and *Pain and Glory* depict the use of the arts to help with coping with a life with pain. Frida Kahlo is shown in various scenes painting in her bed, and throughout the film her pursuit of art is seen as a way for her to cope with her pain. Even others see therapy in her art; Trotsky confides this to her: “That’s what I loved about your paintings. That they carry that message. You said that nobody would care about them, but I think you’re wrong. Because your paintings express what everyone feels. That they are alone, in pain” (1:30:43). Salvador’s reconnection with directing films at the end of the film seems to provide him with a renewed sense of purpose.

## Discussion

This is the first study to review films featuring characters with chronic pain and explore how those characters and their pain management are portrayed. The films reviewed depict the lived experience of characters with chronic pain and how their pain affects their relationships and interactions with others, their experiences with health care professionals, and the management or mismanagement of their pain. Because the films reviewed have been and are easily accessible to the public and many of them have received significant accolades and public attention, there is the potential that these portrayals can influence the public perception of both patients with chronic pain and its management. That possibility lends itself to the use of such films to help with the education of health care professionals regarding chronic pain.

## Depiction of Characters with Chronic Pain

In some ways, the portrayal of characters’ challenges of living with chronic pain in the films reviewed bears semblance to the lived experiences of patients. This is especially the case in films like *Cake* and *Pain and Glory* in which the audience follows Claire and Salvador and witnesses how they struggle with their instrumental activities of daily living and sees how their “lifeworlds” have shrunk.^40(p1107)^ Cinematic depictions of life with chronic pain have been well received by patients,^41^ although it is not clear whether realistic portrayals of illness in film best decrease the stigma that may be associated with them.^42,43^ Röhm and colleagues^44^ suggested that this may be the case because realistic portrayals may disturb viewers such that they then distance themselves even further from them.

In other ways, the portrayal of pain in film was done in a manner that reinforces certain stereotypes of patients with chronic pain. The evidence brief for developing a Canadian national pain strategy recognized that stigma related to chronic pain is a burden,^45^ and a variety of studies demonstrate how common it is.^46–49^ Previous studies that described secondary gain associated with chronic pain^48,50,51^ or the “pain-prone personality”^51(p1461),52^ serve to feed a stereotype that patients with chronic pain are “complainers” or “difficult,”^53(pE204)^ “demanding,” and “manipulative” patients who cannot be trusted.^46,54(p12),55^ A sense of this stigma is apparent in *Cake*, for example, as Claire is seen as challenging to engage with by many of the people in her life and manipulative in terms of her pursuit of opioids. Stigma is thought to influence how patients with chronic pain communicate with others about their pain.^56,57^ Kempner^56^ and Pryma^57^ described how pain is interpreted by people through different lenses and this affects the way it is diagnosed and treated. As a result of stigma, patients may feel the need to present themselves in certain ways to gain credibility,^56–58^ as was apparent in *Cake*.

## Depiction of Health Care Professionals’ Attitudes

Studies suggest that patients with chronic pain seek and value their physicians’ interest, empathy, and validation of their pain experiences.^59–66^ The physician in *Cake* does not seem to be attentive to Claire’s needs. An identified barrier to effective pain management is physician attitudes that are contrary to those ideals; patients have expressed that they have felt blamed, dehumanized, and judged by physicians when they present for management of chronic pain.^62,67^ Physicians are not immune to biases and stigma that contribute to patients’ concerns about their pain management.^68–71^ Another barrier is poor physician communication leading to incongruity between patients’ expectations for pain assessment and management and those of their physicians.^59,72–76^

In some ways, then, the resignation that some of the characters in the films reviewed express toward interacting with physicians is not surprising. Salvador and Frida express a resignation toward consulting with physicians. Claire similarly looks beyond her physicians for pain management, traveling with Silvana to Tijuana. There are few and only short scenes with physicians participating in pain management in *Cake* and *Pain and Glory*, potentially giving the audience the sense that the characters do not have much faith in the ability of the physicians to help them. Studies have shown that patients may have trepidation about seeking clinicians’ help with pain management to avoid “bother[ing]” them or because of a “defeatist-type attitude” toward pain management.^77(p12)^ Moreover, studies report a “hostility, antagonism and discomfort” that physicians and patients describe when liaising about chronic pain issues.^78(p840),79^ Diamond and Grauer^80^ found that physicians may find managing chronic pain to be frustrating because the lack of a cure and the challenges associated with managing it can cause them to feel uncertain, overburdened, and inadequate. It is not surprising, then, that studies have found that physicians express dissatisfaction, stress, and low confidence in managing chronic pain.^81–85^

In contrast, many of the depictions of nurses in the reviewed films were complimentary of their role in pain management. Nurses are recognized to have instrumental roles in providing pain care, especially given the amount of time they spend providing direct patient care.^77,86,87^ Studies suggest that, as is the case for physicians, nurses’ biases and attitudes do influence how they assess and manage chronic pain^77,86,88^ and that pain education is important to addressing them.^87,89,90^

Pharmacists are also known to play an important role as part of the multidisciplinary team that helps with chronic pain management, and interactions that patients have with their pharmacists influence the quality of their care.^91–94^ This has been especially clear during the COVID-19 pandemic in which limitations to patients’ abilities to physically attend pain clinics meant that pharmacists played a larger role in monitoring for opioid aberrant behaviors and liaising with physicians about potential concerns about opioid misuse.^95,96^ Pharmacists were rarely depicted in the films reviewed, however. The only depiction occurred in *Cake*, and in that film the pharmacist acts unethically. This portrayal is consistent with Yanicak and colleagues’^36^ finding that the portrayal of pharmacists in American films or television shows has been typically negative.

## Depiction of Pain Management Strategies

The negative light in which pharmacological pain management strategies like opioids are depicted at times in the reviewed movies speaks to the stigma that can be associated with their use. Systematic reviews suggest that opioids may only provide small benefit for chronic pain.^97^ In some films, it seems to be a treatment of last resort, as is the case in *Pain and Glory*, in which characters with pain use heroin when it seems there are few other options available to them. In other cases, its use is seen to be fraught with problems, as is seen in *Cake*, where Claire demonstrates a number of opioid aberrant behaviors and her opioid use can be argued to contribute to the development of her hallucinations. In other films, opioid use is seen to be associated with succumbing to illness or suffering, as is seen in *The English Patient*. Depiction of opioid use in this negative light is consistent with literature suggesting that patients who use opioids to manage their pain experience stigma related to its use, including being considered “morally weak” or drug-seeking, and thus encounter numerous barriers to receiving such therapies.^55,94(p98–100),128^ According to clinical practice guidelines, various characters’ use of opioids for pain management would merit their clinicians’ attention and reconsideration of the use of opioids for pain management as well as their consideration of treatment of opioid use disorder.^101^

*The Upside* is the only movie among the ones reviewed that included scenes related to cannabis use for pain. This film is also unique because Hollywood films typically depict recreational and not therapeutic use of cannabis, as exemplified by films such as *Harold & Kumar Go to White Castle, Knocked Up*, and *Pineapple Express*. One recent systematic review found that the evidence from randomized controlled trials for the use of cannabis for chronic pain management was of low or very low quality and few beneficial effects have been found,^102^ whereas another found that the use of noninhaled cannabinoids resulted in only a small to very small improvement in pain relief.^103^ Despite the weakness of this evidence, offering Philip from *The Upside* a trial of noninhaled cannabis or cannabinoids as an adjunct therapy given that his pain management is insufficient would be supported by a recently published clinical guideline.^104^ Studies suggest that patients with pain have the perception that their use of cannabis is becoming increasingly supported by their health care providers,^105^ although stigma regarding its use still exists.^105–107^

None of the characters in the films reviewed are seen as receiving multidisciplinary care of their pain. The closest to this is seen in *Cake*, where Claire accesses the care of a physician, physiotherapist, and pain workshop facilitator. However, this occurs in a completely fragmented manner.

## Implications for Education

The content analysis performed in this study demonstrates that there is diverse content in contemporary feature films related to the depictions of characters with chronic pain and their management, and we suggest that these depictions could be used in a cinemeducation approach to teaching trainees about chronic pain management. After learners watch one or more of these reviewed films, their reflections could be used as springboards for further discussion with their clinical teachers about chronic pain management. For example, review of film clips from *Cake* could be used in discussions about how health care professionals can either positively or negatively contribute to patients’ pain care. *Pain and Glory* could be used to provide greater perspective to learners about the complex psychosocial factors that influence patients’ pain experiences. Indeed, each of the themes found in our review lend themselves to topics of discussion with learners, and the films could be used to provide context for that.

To date, the use of cinemeducation as a tool in chronic pain management education has not been described. Cinemeducation has been used in educational curricula in family medicine,^108,109^ palliative care,^110,111^ medical ethics,^112–114^ and psychiatry.^115–121^ Films are particularly amenable to incorporation in medical school and residency curricula.^122^ Clips or entire films can be used to bring case studies to life^123^ so that rather than reading or hearing about a case, trainees are provided with a fuller vision of the way illness is experienced.^124^ One can also see how illness unfolds over the course of a film, providing a more longitudinal case study experience. The viewing of films in medical education has been compared to the usual practice in medicine of observing and listening to patients and then reflecting upon that information.^122^ Shapiro and Rucker^125^ described a “Don Quixote” effect in which the viewing of cinematic clinical scenarios can evoke a greater emotional and empathic reaction than experiencing the encounter itself. Kumagai^126^ suggested that learning opportunities based upon narratives and the humanities offer a “fundamentally different”^(p656)^ education compared to typical biomedical teaching in that they are “transformative”^(p656)^ by “allowing glimpses into the subjective world of lived experience, forging emotional links with the other, stimulating self-reflection through cognitive dissonance, and eliciting resonance of similar, fundamental emotions in the learner.”^(p657)^

## Limitations

It is possible that not all relevant films were found, because, as Auwen and colleagues noted,^2^ key words in a database like IMDb may change over time. Moreover, the films selected for review in this study were produced by the North American film industry or were deemed to have the potential to have influence on popular culture as evidenced by the receipt of significant awards, such as Academy Awards or Golden Globes, or box office revenue. In that regard, this study is biased toward themes that emerge from a predominantly contemporary North American perspective and from cultural significance from a North American–centric lens. Moreover, the films reviewed include characters who might primarily identify in society as White cis-het. Review of films from other countries and cultures would likely provide different depictions and themes related to chronic pain. The importance of engagement in reflexivity in the qualitative research process has been noted.^127^ We acknowledge that our own beliefs and assumptions influence our interpretation of the films. During the coding process, we considered complementary and divergent opinions about the themes. Thus, given the subjective nature of cinema, the themes presented in this study are not exhaustive and other audiences might consider other themes to emerge from analysis of the films.^128^ Nevertheless, the exploratory nature of this study provides the basis for future studies looking in more detail at themes related to pain management in cinema as well as at whether or how films influence societal attitudes toward patients with chronic pain and its management. Work is ongoing to develop film-based learning modules for learners while they are on clinical rotations to give them a better perspective of the biopsychosocial nature of chronic pain.
